# Technology Advances, High-Risk Research, and a Safe Way Forward

**DOI:** 10.1128/mBio.02373-21

**Published:** 2021-10-26

**Authors:** David R. Franz, James W. Le Duc

**Affiliations:** a Former commander, U.S. Army Medical Research Institute of Infectious Diseases, Frederick, Maryland, USA; b Former director of the Galveston National Laboratory, University of Texas Medical Branch, Galveston, Texas, USA

**Keywords:** advanced technologies, high-risk research, leadership, nuanced regulation

## Abstract

The human and economic toll of the coronavirus disease 2019 (COVID-19) pandemic and the unknowns regarding the origins of the virus, with a backdrop of enormous advances in technologies and human understanding of molecular virology, have raised global concerns about the safety of the legitimate infectious disease research enterprise. We acknowledge the safety and security risks resulting from the broad availability of tools and knowledge, tools and knowledge that can be exploited equally for good or harm. The last 2 decades have shown us that the risks are real. They have also shown us that more traditional top-down regulations alone are not the answer. We encourage government to be thoughtful and nuanced in dealing with this significant challenge and to carefully consider human factors and the important role of organizational-level leadership before simply layering an additional bureaucratic burden on the enterprise without understanding value and cost.

## COMMENTARY

Uncertainties still surround the origins of the coronavirus disease 2019 (COVID-19) pandemic, which is believed to have sickened more than 200 million, killed more than 4 million, and terribly disrupted economies worldwide. The pandemic continues to rage, now with variant viruses and in third and fourth waves. A mix of responsible and irresponsible journalism and political bias confuse and even divide populations with simplistic explanations focusing on “lab leak” or “jump from nature.” Some suggest the possibility of laboratory manipulations of the virus. One of the expected calls, among the many, is for more regulation of our own infectious disease life sciences enterprise.

We in the United States have a multiple-decade history of high-containment infectious disease research without catastrophic outcomes. We have responded to several minor and even potential safety and security incidents—resulting from carelessness, poor judgment, and even crime—with crude bureaucratic regulatory tools that often negatively impact the vast majority who are careful scientists. We do not have good metrics for regulatory effectiveness; however, the absence of laboratory-acquired disease and the lack of serious breaches of biocontainment attest to the inherent safety of modern U.S. high-containment facilities. Not surprisingly, the advanced knowledge and technologies available in our laboratories today are equally powerful in the hands of “good” and “bad” scientists, competent and incompetent. We do not doubt the potential for great harm. Thus, we need to be agile in adapting to new potential risks associated with technological advances while maintaining our ability to make the scientific progress that clearly benefits society.

It is not that we have not considered surprises from nature and even from our labs. We adopted the Select Agent Rule nationally in 1996 and expanded it in 2002 all in response to “security” incidents. When there were few new security incidents, we made lab safety the focus. In 2004, after 9/11 and the “anthrax letters” wake-up calls, the National Academies of Sciences, Engineering, and Medicine (NASEM) published the “Fink report,” *Biotechnology Research in an Age of Terrorism* (1), which gave us common-sense examples of the kinds of harm that could come from our research enterprise if we are not watchful and careful. While an important first step in developing our thinking, it took us down the road of “dual-use research of concern,” highlighting the technologies more than the human behavior, typically the cause of “misuse” when it does occur. The Fink report recommended that a national board be established which would serve as an advisory body on these matters. In 2005, the National Science Advisory Board for Biosecurity (NSABB) was put in place and quickly dealt with security questions related to the publication of the sequence of the recovered 1918 pandemic flu strain. In 2007, the NSABB Framework for Oversight of Dual Use Life Science Research (2) established guidelines for evaluating potentially dangerous research “from hypothesis to publication.” In 2008, the Department of Army borrowed from the nuclear enterprise to establish a Surety Program (Safety, Security, Agent accountability and Personnel reliability), some of which is ill-suited to biology. Also, in 2008/2009, four national committees (3) considered how to deal with the “insider threat,” a similar challenge to the life sciences community. Just a few years later, the NIH funded two scientists who successfully (and safely) engineered H5N1 flu virus, making it aerosol transmissible in ferrets; this was high-risk research. The NSABB and international colleagues helped journals deal with the issue of publication of those potentially abusable data, eventually voting to allow it. (Even that exercise was somewhat artificial, because findings from academic research done in a free society like ours cannot really be “put back in the bottle.”) In 2016, the NSABB published *Recommendations for the Evaluation and Oversight of Proposed Gain-of-Function Research* (4), considering the period from “research design to approval for funding.” Then, in 2017, the White House Office of Science and Technology Policy crafted *Recommended Policy Guidance for Potential Pandemic Pathogen Care and Oversight* (5) based on the NSABB recommendations and established a Department of Health and Human Services (DHHS) committee to review and manage such potentially risky research. Questions regarding the diligence with which the NIH subsequently applied these new oversight tools and the transparency associated with the DHHS review process exist.

To date, much of the effort to minimize the risk of misuse of modern technology and its information product has been undertaken by journals and shared by the researchers themselves. While an important piece of the puzzle’s solution, their impact occurs primarily after the research has been done and is further limited to consideration of what is deemed appropriate for sharing by the researchers. While it is important for publishers to take careful note of dual-use research of concern (DURC) issues, their engagement is far too late in the process. The guidelines from the NSABB in 2007 and 2016/2017 make it clear that the early period around hypothesis development is where oversight should begin. That will require organizational leadership with integrity and a strong sense of responsibility, which is then embraced by all involved, from students and technicians to principal investigators (PIs) and program leaders. If done well, many proposals to conduct “dangerous research” will never even make it to a NIH study section.

Now, with the COVID-19 pandemic swirling around us all, political scientists, arms control experts, and some biologists, many of whom have never served in a high-containment laboratory, are calling for more regulation of the enterprise. Others just want the U.S. government, or even international bodies, to crack down on what they call “gain-of-function” research. What is needed? What will work? What will be the costs? Some, having observed the bureaucratic implementation of the Select Agent Rule and other regulations, call for nuance by the government. But is the government capable of nuance?

We have seen detailed lists of technologies for which there is risk of harm, although the vast majority will be used for good. It is sad that in this era we need to speculate how someone might abuse almost every sector and tool of biotechnology—well beyond the Fink seven deadly sins—for harm. There have even been calls for international regulatory and monitoring mechanisms, to among other things, supervise research on dangerous pathogens.

Leaders of high-containment labs have responsibly managed work with dangerous pathogens for decades. Are we now to assume that where evil can be applied, it will be, unless there is top-down government intervention regarding use of these technologies? We do not believe that more regulations will necessarily equate to a safer future. There will always be sound, responsible leadership and healthy organizational cultures in most high-containment laboratories around the globe, whether there is big government oversight or not. The highest containment level, biosafety level 4 (BSL-4) labs, now more than 50 globally, often have better oversight and management than the much more numerous BSL-3 and BSL-2 labs. Nor are we suggesting that the “potential” for harm from nature or the lab is not enormous. The COVID-19 pandemic has demonstrated the tragic consequences of a novel pathogen on society. We only hope that careful thought is given to both the benefits and the potential unintended consequences that might result from additional regulatory interventions applied at this point in our enterprise history. We must think before we regulate and consider cost and benefit.

We have previously extolled the virtues of sound leadership at the institute level to deal with traditional laboratory safety and security, including the “insider threat” (6). We believe that the primary responsibility for conducting safe and secure research with highly hazardous pathogens broadly should be at the organizational level, accepted as a responsibility by the leadership of the institute or laboratory conducting the work. Near the bench rather than the Beltway! History has shown that successful leaders lead with quality science, emphasis on safety, vision, education, responsibility, accountability, honesty, transparency, and ethics. Such leaders invariably have an open-door policy and build networks of regular and open communication within their organization and with other institutes and their scientists. It is not difficult for such an enlightened and selfless leader to develop an institutional culture of accountability and trust based on those basic principles. It has worked in the past; it will work today. Yet our experience suggests it is becoming progressively more difficult to develop, trust and reward enlightened leaders who just “do the right thing.”

For the most part, there will always be humans with significant understanding of the risks and benefits, and the moral courage, to lead and operate safely in the infectious disease research enterprise no matter where technologies and knowledge take us. But there will also always be some who are careless or thoughtless or those who take excessive risk for their own reasons. Unfortunately, it is apparently easier to add regulations than it is to ensure that humans are responsible for their actions.

Much of the cry is for more regulatory oversight at the bench level, the approach we have taken in the past. This risks making legitimate work more difficult and expensive for the vast majority of the enterprise’s scientists and leaders who do take full responsibility for their actions and publications. Yet, the specific examples we have of the most high-risk research conducted in recent years suggest that “go-no go” decisions are often made at the government funding agency level. The concept of educating scientists about the importance of individual and corporate responsibility and, particularly, further developing sound leaders and a culture of responsible science and expecting excellence from them, will likely be supported by those at the bench who understand…. but what will happen to implement the call from a few for nuanced oversight? The four studies in 2008, mentioned above, made similar proposals supporting “the importance of leadership, cultures of responsibility, accountability, trust, values and moral obligations.” There has been little apparent effort by government to implement those recommendations made more than 10 years ago. Let us hope this time it will be different. New regulations, almost by definition, cost time and money…and can reduce the pool of young scientists interested in the field. Neither regulation nor leadership alone are a 100% solution, but sound leadership “doing the right thing” in our labs costs nothing. Let us do all we can to support a safe, secure, and productive enterprise while not hobbling our researchers unnecessarily with additional burdensome oversight that does little to make society safer but hampers our progress in addressing the disease threats of today and tomorrow. We are in a serious global competition.
